# The Direct Actions of GABA, 2’-Methoxy-6-Methylflavone and General Anaesthetics at β3γ2L GABA_A_ Receptors: Evidence for Receptors with Different Subunit Stoichiometries

**DOI:** 10.1371/journal.pone.0141359

**Published:** 2015-10-23

**Authors:** Han Chow Chua, Nathan L. Absalom, Jane R. Hanrahan, Raja Viswas, Mary Chebib

**Affiliations:** Faculty of Pharmacy, University of Sydney, Sydney, New South Wales, Australia; McLean Hospital/ Harvard Medical School, UNITED STATES

## Abstract

2’-Methoxy-6-methylflavone (2’MeO6MF) is an anxiolytic flavonoid which has been shown to display GABA_A_ receptor (GABA_A_R) β2/3-subunit selectivity, a pharmacological profile similar to that of the general anaesthetic etomidate. Electrophysiological studies suggest that the full agonist action of 2’MeO6MF at α2β3γ2L GABA_A_Rs may mediate the flavonoid’s *in vivo* effects. However, we found variations in the relative efficacy of 2’MeO6MF (2’MeO6MF-elicited current responses normalised to the maximal GABA response) at α2β3γ2L GABA_A_Rs due to the presence of mixed receptor populations. To understand which receptor subpopulation(s) underlie the variations observed, we conducted a systematic investigation of 2’MeO6MF activity at all receptor combinations that could theoretically form (α2, β3, γ2L, α2β3, α2γ2L, β3γ2L and α2β3γ2L) in *Xenopus* oocytes using the two-electrode voltage clamp technique. We found that 2’MeO6MF activated non-α-containing β3γ2L receptors. In an attempt to establish the optimal conditions to express a uniform population of these receptors, we found that varying the relative amounts of β3:γ2L subunit mRNAs resulted in differences in the level of constitutive activity, the GABA concentration-response relationships, and the relative efficacy of 2’MeO6MF activation. Like 2’MeO6MF, general anaesthetics such as etomidate and propofol also showed distinct levels of relative efficacy across different injection ratios. Based on these results, we infer that β3γ2L receptors may form with different subunit stoichiometries, resulting in the complex pharmacology observed across different injection ratios. Moreover, the discovery that GABA and etomidate have direct actions at the α-lacking β3γ2L receptors raises questions about the structural requirements for their respective binding sites at GABA_A_Rs.

## Introduction

γ-aminobutyric acid type A receptors (GABA_A_Rs) are a ubiquitous class of inhibitory receptors with high physiological significance in the central nervous system (CNS). Structurally, GABA_A_Rs are made up of five pseudo-symmetrically arranged subunits surrounding a central anion-conducting pore. While a vast array of receptor combinations could theoretically form from the large number of known GABA_A_R subunits (α1–6, β1–3, γ1–3, δ, ε, θ and π), most native GABA_A_R subtypes are made up of α, β and γ subunits, and to a smaller extent α, β and δ [1]. Receptors of different subunit composition have distinct distribution patterns, physiological roles, as well as electrophysiological and pharmacological properties [2–5].

GABA_A_Rs are the site of action for many clinically-relevant chemicals such as benzodiazepines, barbiturates and anaesthetics. These drugs typically bind to different interfaces between two neighbouring subunits that form the principal (+) and complementary (−) components of their binding sites. In the most common GABA_A_R isoform αβγ with a stoichiometry of 2α:2β:1γ and a counter-clockwise arrangement of γ-β-α-β-α when viewed from the synaptic cleft [6–8], the extracellular domain of the β+α− and the α+γ− interfaces are known to harbour the GABA and benzodiazepine binding sites respectively [9–11]. In contrast, general anaesthetics such as etomidate and propofol have been shown to bind to the transmembrane domain of the β+α− interface [11–13]. High prominence has been given to the β+α− and α+γ− interfaces due to the successful clinical utilisation of GABA_A_R modulators that bind to these interfaces. The pharmaceutical potential of the remaining α+β− and γ+β− interfaces is also an area of active research [14–18].

Heterologously expressing a single defined GABA_A_R subtype to evaluate the structure-activity relationship of lead compounds has become an indispensable component in drug discovery. However, mixed receptor populations are a common problem that contributes to variability between pharmacological studies. For instance, the measurement of diazepam enhancement at αβγ receptors has been shown to vary when contaminated with the diazepam-insensitive αβ receptors [19, 20]. Besides that, the binary αβ receptors have been shown to assemble with two different stoichiometries (2α:3β and 3α:2β), but the functional significance of this finding is not understood [6, 21, 22]. There have also been several independent reports of the robust βγ receptor expression and their responsiveness to GABA and various ligands, including diazepam, implicating binding sites for these ligands other than the traditional β+α− and α+γ− interfaces [23–29]. However, the impact of βγ receptors on the pharmacological studies conducted on αβγ receptors, their potential as a tool receptor to interrogate GABA_A_R pharmacology, as well as their *in vivo* existence have not been explored.

We have previously reported that the synthetic flavonoid 2’-methoxy-6-methylflavone (2’MeO6MF) induced anxiolysis in mouse behavioural studies [30]. In single-channel recording experiments, 2’MeO6MF was found to directly activate native γ2-containing GABA_A_Rs found in rat hippocampal neurons in the absence of GABA. Electrophysiological studies conducted across 18 different GABA_A_R subtypes expressed in *Xenopus* oocytes identified α2β2/3γ2L GABA_A_Rs as the targets for the direct activation of 2’MeO6MF. However, the activity of 2’MeO6MF at βγ receptors was not explored. Experimental evidence from electrophysiological and behavioural studies supports a non-benzodiazepine mechanism for 2’MeO6MF effects. Mutational studies found that the N265 residue on the β2/3 subunits, a key determinant of etomidate’s sensitivity [31–33] is crucial for 2’MeO6MF activity, suggesting that 2’MeO6MF and etomidate may share a similar mechanism of action.

In this study, we found variations in 2’MeO6MF relative efficacy at α2β3γ2L receptors, which we associated with the presence of mixed receptor populations. We systematically expressed all possible receptor combinations in *Xenopus* oocytes using α2, β3 and γ2L subunits and found that the non-α-containing β3+γ2L combination formed robust GABA-gated receptors which were also activated by 2’MeO6MF. Varying the mRNA injection ratio of β3 and γ2L subunits resulted in unique pharmacological profiles for GABA, 2’MeO6MF, etomidate and propofol, most likely due to stoichiometric differences in the receptors being expressed. The α subunit does not appear to be strictly needed for receptor function as demonstrated by the ability of GABA and etomidate to activate β3γ2L receptors. We infer from these findings that the β subunit, which bears the principal (+) components of the respective binding sites dominates ligand binding interactions, and the identity of the complementary (−) subunit (α, β or γ) is well tolerated.

## Materials and Methods

### Reagents

GABA, flumazenil, zinc chloride (ZnCl_2_), DMSO and all buffer ingredients mentioned in this study were purchased from Sigma-Aldrich (St. Louis, MO, USA) while diazepam was purchased from Apin Chemicals LTD (Abingdon, Oxon, UK). Etomidate hydrochloride (HCl) was synthesised following protocols previously described in Janssen *et al*. [34] and Janssen *et al*. [35]. The synthesis of 2’MeO6MF was previously described in Karim *et al*. [30].

### GABA_A_R subunit constructs

Human complementary DNA (cDNA) of GABA_A_R α2 subunit (vector: pCMV6-XL5) was purchased from OriGene Technologies (Rockville, MD, USA), and the human β3 (vector: pGEMHE) and γ2L (vector: pCDM8) subunits were generous gifts from Dr. Bjarke Ebert (H. Lundbeck A/S, Valby, Denmark) and Dr. Paul Whiting (Merck, Sharp and Dohme Research Laboratories, Harlow, Essex, UK) respectively. The identity of the subunits was confirmed with DNA sequencing (Australian Genome Research Facility, Westmead, NSW, Australia). Plasmid vectors with the cDNA incorporated were linearised using appropriate restriction endonucleases (α2: SmaI; β3: NheI; γ2L: NotI). Capped RNA transcripts were synthesised from linearised plasmids using the mMessage mMachine T7 transcript kit from Ambion (Austin, TX, USA). Purified messenger RNA (mRNA) was dissolved in nuclease-free water (Ambion, Austin, TX, USA), and its quality was assessed using 1% agarose gel electrophoresis. The concentration of each subunit mRNA was measured using Nanodrop ND-100 UV-Vis spectrophotometer (Thermo Fisher Scientific, North Ryde, NSW, Australia).

### *Xenopus* oocytes isolation and preparation

Mature female *Xenopus laevis* frogs were anaesthetised with 0.17% tricaine (buffered with 0.06% sodium bicarbonate) for 15 minutes, after which the loss of righting reflex was confirmed before transferring on to ice where surgeries were performed. A small (1–2 cm) abdominal incision was made through both the skin and muscle layer with surgical knives. Ovary lobes were removed with a pair of forceps, and kept in oocyte releasing 2 (OR2) buffer (82.5 mM NaCl, 2 mM KCl, 1 mM MgCl_2_, 5 mM HEPES hemisodium; pH 7.4). The skin and muscle layer were sutured separately, and frogs were allowed to recover for six months before they were reselected for surgeries. A total of five recoverable surgeries were allowed on each frog, before a terminal surgery was performed, in which a lethal dose of tricaine (0.5%) was used. All the procedures involved in the use of *Xenopus laevis* frogs were approved by the Animal Ethics Committee of the University of Sydney (Reference number: 2013/5915). Removed ovary lobes were divided into small sections and treated with 2 mg/mL Collagenase A (Boehringer Mannheim, Indianapolis, IN, USA) in OR2 buffer for at least an hour to defolliculate the oocytes. The released oocytes were then rinsed in ND96 storage solution (96 mM NaCl, 2 mM KCl, 1 mM MgCl_2_, 1.8 mM CaCl_2_, 5 mM HEPES hemisodium, 2.5 mM pyruvate, 0.5 mM theophylline; pH 7.4). Healthy-looking stage V–VI oocytes were isolated and kept in ND96 storage solution at 18°C until ready for injection.

### Electrophysiological recording of recombinant GABA_A_Rs in *Xenopus* oocytes

Various combinations of α2, β3 and γ2L subunit mRNAs were mixed to different ratios as stated in the text, and diluted accordingly to a concentration of 5 ng/μL (unless otherwise stated). The mRNA mixture was injected into the cytoplasm of oocytes (50.6 nL/injection) using a Nanoject device (Drummond Scientific, Broomali, PA, USA). Injected oocytes were stored in ND96 storage solution supplemented with 50 μg/mL gentamycin and tetracycline at 18°C. Two-electrode voltage clamp recordings were performed on oocytes 2–4 days post-injection at room temperature (20–25°C) using a GeneClamp 500B amplifier (ADInstruments, Sydney, NSW, Australia) or a Warner OC-725C Oocyte Clamp (Warner Instrument Corp, Hamden, CT, USA). All experiments were conducted at a holding potential of –60 mV, and data were acquired with a PowerLab 2/25, and LabChart (version 5.0.2) software (ADInstruments, Sydney, NSW, Australia). The recording microelectrodes (0.2–1.1 MΩ) were generated from capillary glass (Harvard Apparatus, Holliston, MA, USA) using a single-stage glass microelectrode puller (Narishige, Tokyo, Japan), and were filled with 3 M KCl solution. The perfusion system was built using a series of polycarbonate, high-density polyethylene two-way stopcocks (Qosina, Edgewood, NY, USA), connected through tubing to the recording chamber (100 μL), where the oocyte was continuously perfused with ND96 recording solution (96 mM NaCl, 2 mM KCl, 1 mM MgCl_2_, 1.8 mM CaCl_2_, 5 mM HEPES hemisodium; pH 7.4) at an approximate rate of 5 mL/min.

GABA, etomidate and ZnCl_2_ were dissolved in ND96 recording solution, whereas 2’MeO6MF, diazepam and flumazenil were dissolved in DMSO to make up stock solutions of desired concentrations. GABA stock solutions were prepared fresh on the day of experiments. When working with chemicals dissolved in DMSO, all perfusates were standardised to contain 0.8% DMSO, which did not produce any alteration in the recordings. Perfusates containing these chemicals were made up fresh before each application. The concentration-response curves of agonists were obtained by exposing oocytes to increasing concentrations of agonists until the currents peaked. Maximal GABA current responses (usually 3 mM, unless otherwise stated) were used as controls to allow for comparisons between different oocytes. Depending on the concentration and the solubility of the chemicals applied on oocytes, the wash-out periods ranged from 3 to 15 minutes to allow time for receptors to recover from desensitisation. The modulatory effect of 2’MeO6MF was also investigated by co-applying a low concentration of GABA (EC_3–10_) with a high concentration of 2’MeO6MF (100 or 300 μM). No enhancement of GABA current was detected at α2β3γ2L (3:1:3), α2β3 (20:1), β3γ2L (1:15) and (1:100) receptors (data not shown). All experiments were performed using at least two different batches of oocytes.

### Data analysis

Prism (version 5.04; GraphPad Software, La Jolla, CA, USA) was used for data analysis. Raw data from GABA concentration-response experiments were fitted to either a monophasic (1) or biphasic (2) Hill equation, and the fitted maximal values from the preferred model (Extra sum-of-squares *F* test) were used for normalisation of each dataset.

$$I=\frac{I_{\text{max}}}{1+10^{\left(({\text{logEC}}_{50}-[A])n_{\text{H}}\right)}}$$ (1)

$$I=\frac{I_{\text{max}}\times \text{Frac}}{1+10^{\left(({\text{logEC}}_{50\_1}-[A])n_{\text{H}\_1}\right)}}+\frac{I_{\text{max}}\times (1-\text{Frac})}{1+10^{\left(({\text{logEC}}_{50\_2}-[A])n_{\text{H}\_2}\right)}}$$ (2)

Concentration-response curves of other agonists such as 2’MeO6MF and etomidate were constructed in a similar manner, except the data were normalised to the 3 mM GABA responses, unless otherwise stated. In these equations, *I*_max_ represents the maximal agonist response, [*A*] represents the agonist concentration, EC_50_ represents the agonist concentration required to activate 50% of the maximal response (there are two EC_50_s for a biphasic curve), *n*_H_ represents the Hill slope of the fitted curve (there are two Hill slopes for a biphasic curve), and Frac represents the proportion of the maximal response mediated by the more potent component in a biphasic curve. In all cases, we constrained the bottom to 0, and when fitting data to the biphasic model, the Hill slopes were constrained to 1. Statistical comparisons of results for different injection ratios of β3γ2L receptors were performed using ANOVA with Tukey’s *post-hoc* test. When comparing results from different injection ratios of β3γ2L receptors with α2β3γ2L receptors, the ANOVA Dunnett’s test was used. Pairwise comparisons were performed using Student’s *t* test. Statistical significance was attained at *p* < 0.05.

## Results

### Variations in the relative efficacy of 2’MeO6MF activation at α2β3γ2L GABA_A_Rs

**Fig 1:**
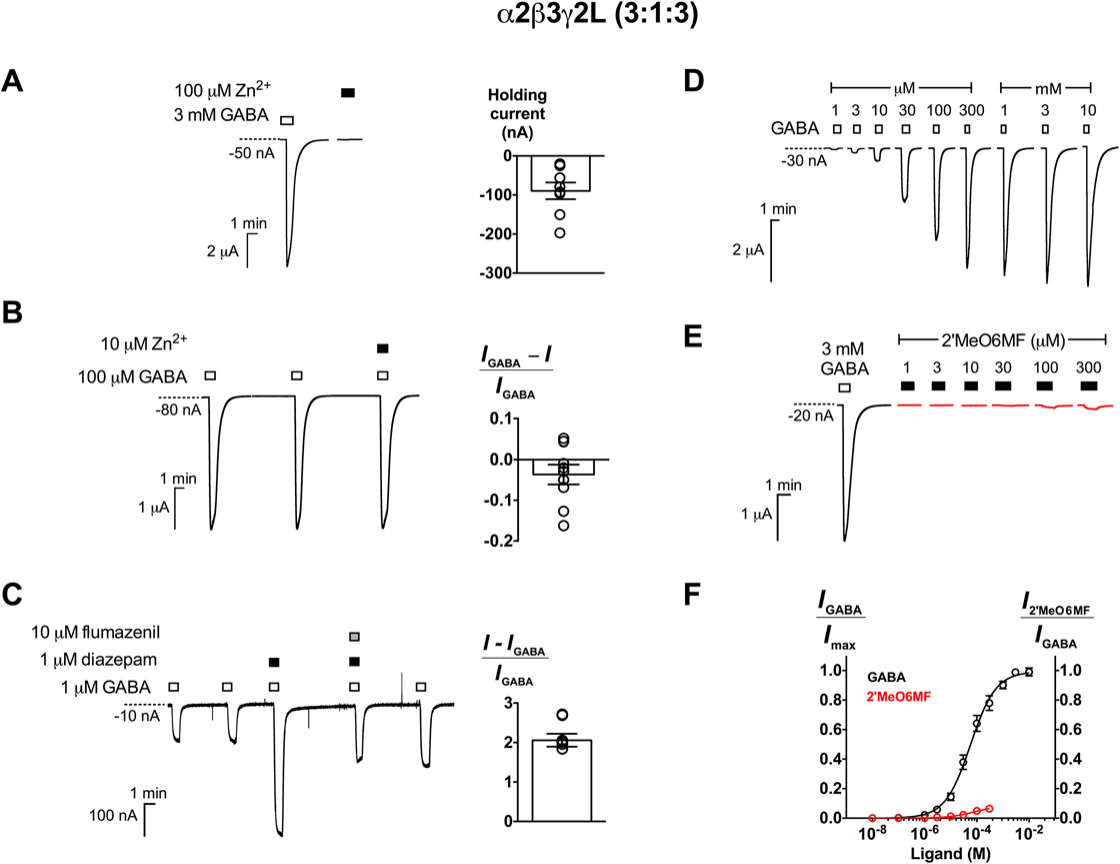
Characterisation of α2β3γ2L GABA_A_Rs expressed at a 3:1:3 injection ratio. (A) *Left panel*, Representative traces of α2β3γ2L (3:1:3) GABA_A_Rs responses to 3 mM GABA and 100 μM Zn^2+^ alone. *Right panel*, Mean holding current of oocytes expressing α2β3γ2L (3:1:3) GABA_A_Rs (-93 ± 19 nA; *n* = 8). (B) *Left panel*, Continuous traces demonstrating two consecutive applications of control (100 μM GABA) followed by the co-application of 10 μM Zn^2+^ with control. *Right panel*, Modulation of 100 μM GABA responses by 10 μM Zn^2+^ (*n* = 9). (C) *Left panel*, Continuous traces demonstrating two consecutive applications of control (1 μM GABA) followed by the co-application of 1 μM diazepam with control; 1 μM diazepam and 10 μM flumazenil with control; and control. *Right panel*, Potentiation by 1 μM diazepam of 1 μM GABA responses (*n* = 6). Representative traces demonstrating (D) GABA current responses from 1 μM to 10 mM and (E) 2’MeO6MF’s direct activation from 1 to 300 μM (red) in comparison to 3 mM GABA response. Concentration-response curves of GABA (black; *n* = 6) and 2’MeO6MF (red; *n* = 5) are shown in (F). Data are presented as mean ± SEM. Bars indicate durations of drug application. The holding current values are represented by the dotted lines.

2’MeO6MF has been previously described as a full agonist at α2β3γ2L GABA_A_Rs expressed at a 1:1:10 injection ratio in *Xenopus* oocytes [30]. We expressed α2β3γ2L receptors at a 3:1:3 injection ratio and recorded pharmacological responses to GABA, Zn^2+^, diazepam (1 μM) and flumazenil (10 μM) that were consistent with those previously reported (Fig 1 and Table 1) [36–38]. In contrast to what was reported by Karim *et al*. (2012) [30], we found that 300 μM 2’MeO6MF only activated α2β3γ2L receptors 8.2 ± 3.6% of the 3 mM GABA response (*n* = 5; Fig 1E and 1F). The EC_50_ of 2’MeO6MF was estimated to be approximately 74 μM (95% CI: 6.4–850 μM). We hypothesised that the variations in the relative efficacy of 2’MeO6MF measured at α2β3γ2L receptors expressed at different ratios may be due to the presence of mixed receptor populations.

**Table 1:** Characteristics of receptors expressed in various combinations of α2, β3 and γ2L GABA_A_R subunits.

Subunit combination	Ratio	mRNA amount (ng/oocyte)	Expression level (nA)^a^	Holding current (nA)	*n*	2’MeO6MF activity	*n*
**α2**	―	28–35	No current	-20 ± 7.5	8	No current	5
**β3**	―	5	290 ± 67	-1000 ± 420	6	Inverse agonism	5
**γ2L**	―	20–35	No current	-370 ± 41	7	No current	5
**α2β3**	20:1	5	1100 ± 220	-30 ± 7.0	7	Insignificant activation	5
**α2γ2L**	1:1	5	No current	-42 ± 16	4	No current	4
**β3γ2L**	1:1	5	110 ± 34	-470 ± 88	4	Inverse agonism	4
	1:5	5	270 ± 80	-220 ± 60	5	Inverse agonism/activation	5
	1:10	5	310 ± 61	-150 ± 13	8	Inverse agonism/activation	8
	1:15	5	490 ± 47	-260 ± 40	30	Activation	30
	1:20	5	860 ± 87	-180 ± 31	49	Activation	49
	1:50	5	1000 ± 160	-28 ± 6.0	15	Activation	15
	1:100	5	1100 ± 100	-15 ± 5.0	21	Activation	21
**α2β3γ2L**	3:1:3	5	4300 ± 940	-93 ± 19	8	Activation	5

^*a*^ Expression level is expressed as the current responses ± SEM (nA) elicited by 3 mM GABA, except for homomeric and α2γ2L receptors (60 mM GABA).

### 2’MeO6MF does not activate binary α2β3 or homomeric α2, β3 and γ2L GABA_A_Rs

**Fig 2:**
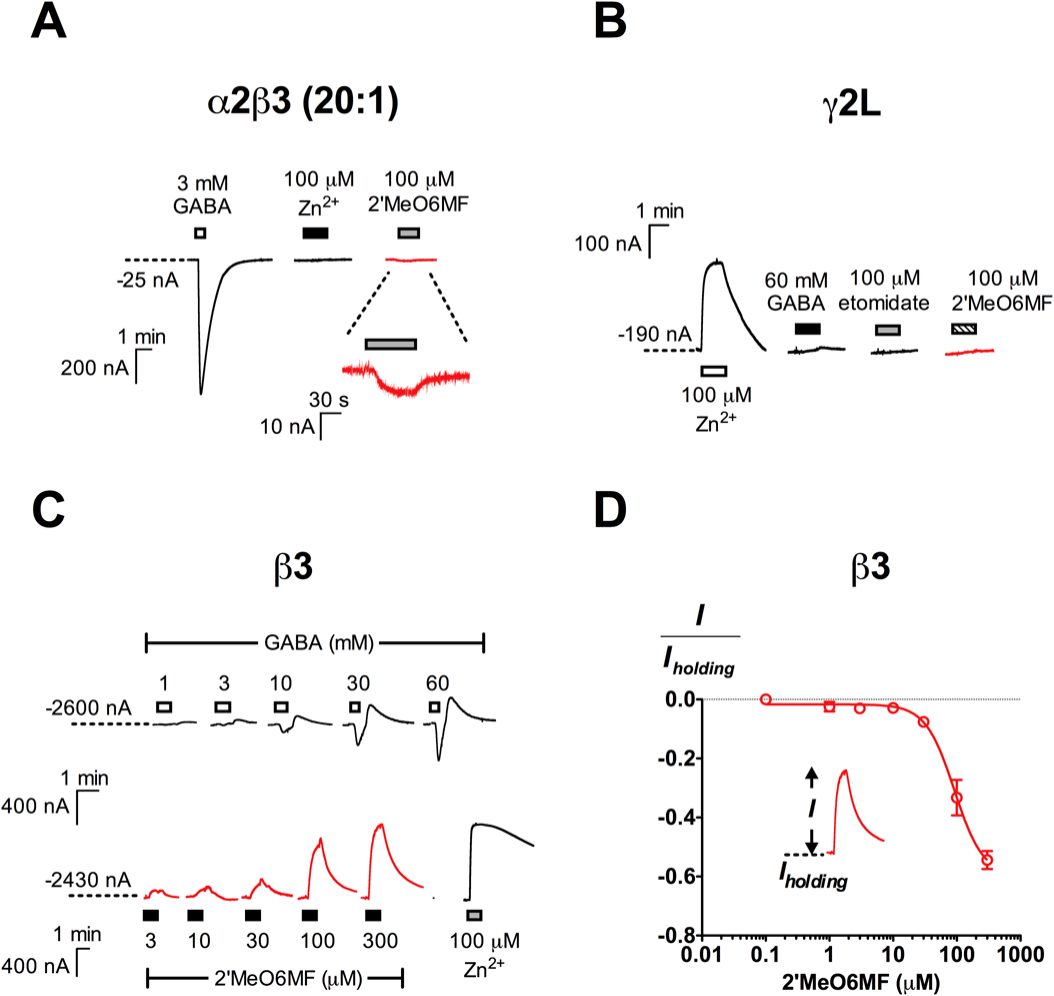
Subunit combinations with detectable function but were not activated by 2’MeO6MF. (A) Representative traces illustrating the robust response elicited by 3 mM GABA, and the lack of activity of 100 μM Zn^2+^ and 100 μM 2’MeO6MF at α2β3 (20:1) GABA_A_Rs (*n* = 6). (B) The injection of γ2L mRNA at high amounts (28–35 ng/oocyte) resulted in constitutively active channels which were inhibited by 100 μM Zn^2+^, but were not sensitive to 60 mM GABA, 100 μM etomidate and 100 μM 2’MeO6MF (*n* = 5). (C) The injection of β3 mRNA (5 ng/oocyte) resulted in the formation of functional receptors. Representative traces of β3 homomeric receptors responses to 1–60 mM GABA (*top*;), 3–300 μM of 2’MeO6MF and 100 μM Zn^2+^ (*bottom*). (D) 2’MeO6MF concentration-response curve for β3 homomeric receptors (*n* = 5). The efficacy of 2’MeO6MF as an inverse agonist is expressed as a fraction of the inhibited spontaneous current (*I*) normalised against the holding current (*I*_*holding*_). Data are presented as mean ± SEM. Bars indicate durations of drug application. The holding current values are represented by the dotted lines.

The presence of αβ receptors when expressing αβγ receptors has been shown to result in submaximal measurement of diazepam modulation [19, 20] as these receptors lack the α+γ− interface where diazepam binds. To determine if this was the cause for differences in 2’MeO6MF relative efficacy at α2β3γ2L receptors, we expressed α2:β3 subunit mRNAs at a 20:1 ratio to prevent β3 homomeric receptors from forming, and found that 2’MeO6MF (100 μM) had negligible activity at these receptors, eliciting currents only 1.7 ± 0.35% of the 3 mM GABA responses (*n* = 5; Fig 2A). Therefore, α2β3 receptors are unlikely to contribute to the much higher 2’MeO6MF relative efficacy previously reported at α2β3γ2L GABA_A_Rs.

To determine if homomeric GABA_A_Rs are activated by 2’MeO6MF, we injected α2, β3 and γ2L mRNAs alone and examined the effect of 2’MeO6MF at these receptors. We did not detect function with 60 mM GABA and 100 μM 2’MeO6MF in *Xenopus* oocytes injected with α2 mRNA (*n* = 5; Table 1). γ2L homomeric receptors showed Zn^2+^-sensitive constitutive activity, but were not responsive to 60 mM GABA and 100 μM 2’MeO6MF (*n* = 5; Fig 2B and Table 1). Consistent with previous findings [39, 40], the injection of β3 subunit mRNA resulted in the expression of constitutively active receptors, which are activated by high concentrations of GABA (Fig 2C and Table 1). In contrast to the direct activation observed at α2β3γ2L GABA_A_Rs, 2’MeO6MF inhibited the constitutive activity of β3 homomeric receptors in a concentration-dependent manner, with a maximal inhibition of 54 ± 3.0% (normalised against oocyte holding current) measured at 300 μM 2’MeO6MF (*n* = 5; Fig 2C and 2D). The lack of activation by 2’MeO6MF at homomeric receptors demonstrates that these receptors are unlikely to be the cause for the variations observed.

### 2’MeO6MF activates β3γ2L GABA_A_Rs with variable efficacy

**Fig 3:**
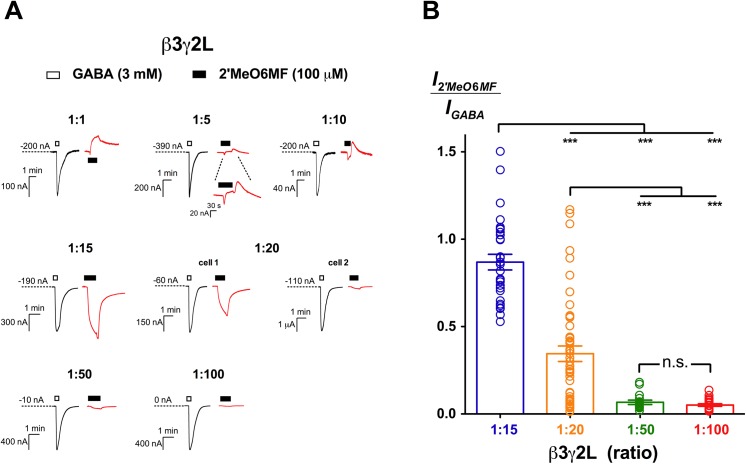
2’MeO6MF activity at β3γ2L GABA_A_Rs. (A) 2’MeO6MF exhibits a complex spectrum of activity across β3γ2L GABA_A_Rs expressed at various injection ratios. Representative traces of 3 mM GABA (black) and 100 μM 2’MeO6MF (red) are shown for each ratio. Bars indicate durations of drug application. The holding current values are represented by the dotted lines. (**1:1**; *n* = 7), 2’MeO6MF inhibited the constitutive activity of receptors expressed. (**1:5**; *n* = 5) and (**1:10**; *n* = 10), 2’MeO6MF exhibited mixed agonist and inverse agonist activity. (**1:15**; *n* = 30), 2’MeO6MF directly activated receptors expressed efficaciously. (**1:20**; *n* = 49), 2’MeO6MF activated β3γ2L (1:20) receptors with variable efficacy. Sample traces of cell 1 and 2 were taken from a simultaneous experiment conducted on two different oocytes injected at the same time. (**1:50**; *n* = 15) and (**1:100**; *n* = 21), 2’MeO6MF showed activation with low efficacy at these receptors. (B**)** Mean efficacy of 100 μM 2’MeO6MF direct activation at β3γ2L (1:15), (1:20), (1:50) and (1:100) GABA_A_Rs. Data are normalised to the 3 mM GABA response. The mean efficacy of 100 μM 2’MeO6MF at various ratios was compared using Tukey’s test, and the significance levels are indicated with n.s. (not significant) and *** (*p* ≤ 0.001).

We then determined whether binary α2γ2L or β3γ2L receptors are activated by 2’MeO6MF. Binary α2γ2L receptors showed no function to 60 mM GABA, 100 μM Zn^2+^ and 100 μM 2’MeO6MF (*n* = 4; Table 1). In contrast, injection of β3 and γ2L mRNAs at a 1:20 ratio to prevent the expression of β3 homomers yielded receptors that responded to GABA. 2’MeO6MF efficaciously activated these receptors, with 100 μM eliciting currents approximately 34 ± 4.0% of the 3 mM GABA responses (*n* = 49; Fig 3). Notably, there was a high variability in the relative efficacy of 2’MeO6MF, ranging from as low as 1.0 to 120% of the 3 mM GABA responses. While the standard error of mean efficacy was only 4.0%, the standard deviation (SD) remained high (31%). This variability was observed both between different batches of oocytes and within the same batch. The substantial variability in the relative efficacy of 2’MeO6MF suggests that the 1:20 ratio was not optimal for the uniform expression of β3γ2L receptors.

To establish the optimal ratio(s) for the uniform expression of β3γ2L GABA_A_Rs, we varied the β3:γ2L mRNAs ratio (1:1, 1:5, 1:10, 1:15, 1:50 and 1:100) while keeping the total amount of mRNA constant at 5 ng in each oocyte. We then measured the relative efficacy of 100 μM 2’MeO6MF and compared it across these receptors. At 1:1 ratio, β3γ2L receptors were constitutively active and 2’MeO6MF acted as an inverse agonist (Fig 3A). This is likely due to the presence of a substantial amount of β3 homomer subpopulation. At 1:5 and 1:10 ratios, where the relative amounts of γ2L mRNA were greater than β3 mRNA, 2’MeO6MF displayed a mixed inverse agonist and agonist effect (Fig 3A), indicating that the pharmacological responses of 2’MeO6MF observed at these ratios were still confounded by β3 homomers.

We increased the injection ratio to 1:15, and consistently observed activation with 100 μM 2’MeO6MF (Fig 3A). The 2’MeO6MF-elicited currents were 87 ± 4.5% of the 3 mM GABA current responses (*n* = 30), which was significantly higher than that at 1:20 ratio (*p* ≤ 0.001; Tukey’s test; Fig 3B). While the low-efficacy component of 2’MeO6MF was not observed at 1:15 ratio, there was still considerable variability in the relative efficacy measured (SD = 25%). Increasing the injection ratios further to 1:50 and 1:100 abolished the high-efficacy component of 2’MeO6MF. 100 μM 2’MeO6MF showed efficacy around 6.7 ± 1.2% (*n* = 15) at 1:50 ratio and 5.1 ± 0.72% (*n* = 21) at 1:100 ratio, which is significantly lower than at the 1:15 ratio (*p* ≤ 0.001; Tukey’s test; Fig 3B). The relative efficacy of 2’MeO6MF measured at α2β3γ2L receptors was similar to that of β3γ2L receptors expressed at 1:50 and 1:100 ratios (*p* > 0.5; Dunnett’s test), but was significantly lower than 1:15 (*p* ≤ 0.001) and 1:20 (*p* ≤ 0.5) ratios.

The extensive variability in 2’MeO6MF relative efficacy measured at 1:20 ratio was likely to be the result of mixed stoichiometries being expressed. This is further supported by variability observed in the GABA concentration-response relationships at β3γ2L (1:20) receptors, in which out of 13 datasets, 6 were better fitted to a biphasic Hill equation, whereas a simpler monophasic model was preferred to describe the rest of the datasets (data not shown). Under our conditions, the 1:15, 1:50 and 1:100 ratios were most optimal to express the different stoichiometries of β3γ2L receptors, allowing us to measure 2’MeO6MF responses more reliably. As such, these ratios were chosen for detailed pharmacological characterisation.

### β3γ2L GABA_A_Rs of different subunit stoichiometries vary in pharmacological properties

**Fig 4:**
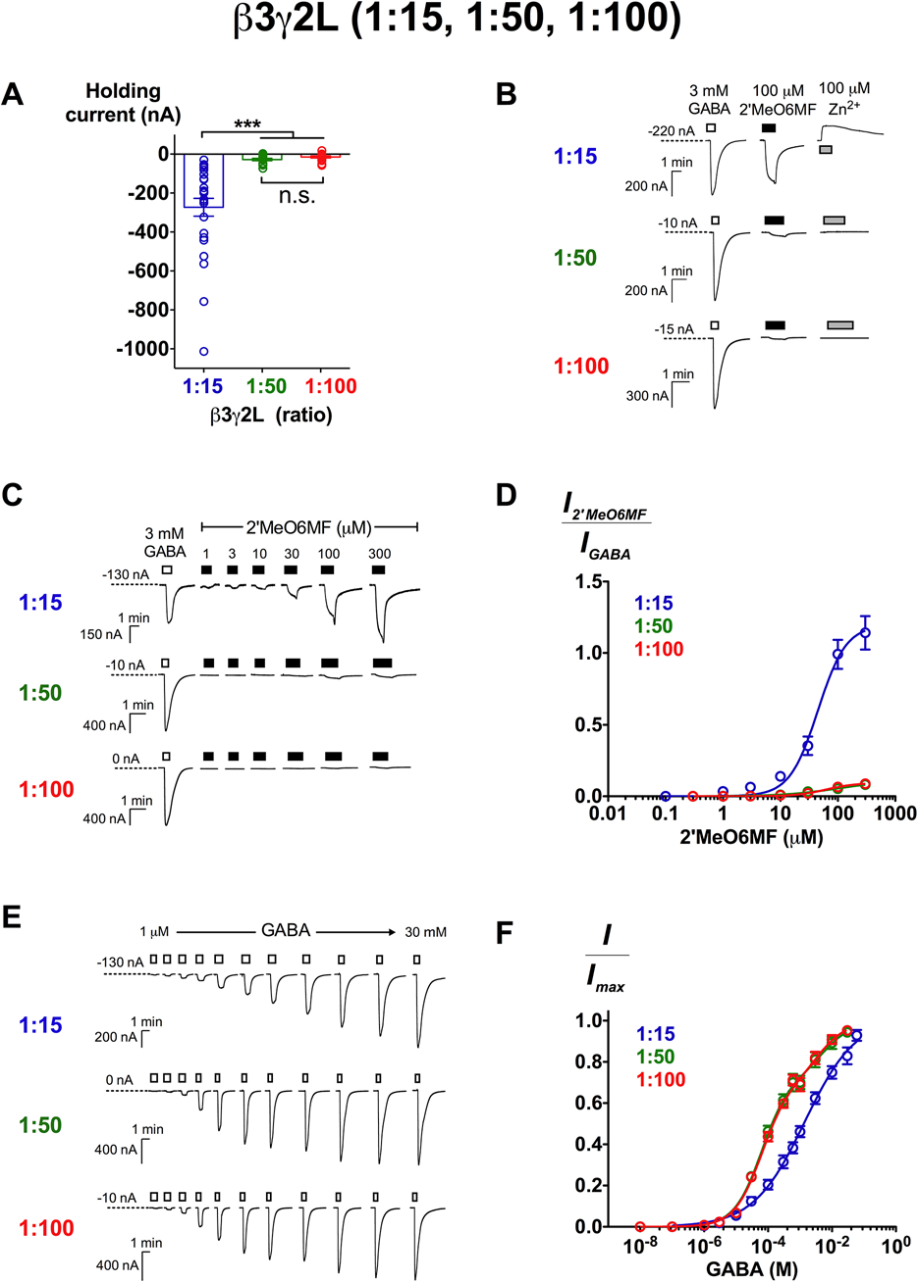
Characterisation of β3γ2L GABA_A_Rs expressed at 1:15, 1:50 and 1:100 ratios. The level of constitutive activity is indicated by (A) holding current of injected oocytes and (B) the inhibition of baseline current by 100 μM Zn^2+^. (A) β3γ2L GABA_A_Rs expressed at 1:15 ratio showed significantly larger holding current (-260 ± 40 nA; *n* = 30) than at 1:50 (-28 ± 6.0 nA; *n* = 15) and 1:100 (-15 ± 5.0 nA; *n* = 21) ratios (*p* ≤ 0.001; Tukey’s test). (B**)** Representative traces demonstrating current responses of 3 mM GABA, 100 μM 2’MeO6MF and 100 μM Zn^2+^. At 1:15 ratio, receptors were sensitive to the inhibition of 100 μM Zn^2+^ (reduction in inward current; *n* = 10). Zn^2+^ did not have any effects at 1:50 (*n* = 7) and 1:100 (*n* = 8) ratios. (C) Representative traces demonstrating 2’MeO6MF’s direct activation from 1 to 300 μM in comparison to 3 mM GABA response. (D) 2’MeO6MF concentration-response curves of β3γ2L (1:15; *n* = 8), (1:50; *n* = 7) and (1:100; *n* = 6) GABA_A_Rs. Data are normalised to the 3 mM GABA response. (E) Representative traces of GABA current responses from 1 μM to 30 mM at β3γ2L (1:15), (1:50) and (1:100) GABA_A_Rs. (F) GABA concentration-response curves of β3γ2L (1:15; *n* = 6), (1:50; *n* = 8) and (1:100; *n* = 9) GABA_A_Rs. Data are presented as mean ± SEM. Bars indicate durations of drug application. The holding current values are represented by the dotted lines.

To obtain an understanding of the properties of different β3γ2L receptor stoichiometries, we assessed constitutive activity, 2’MeO6MF and GABA potencies of the receptors expressed at 1:15, 1:50 and 1:100 injection ratios. Oocytes expressing β3γ2L (1:15) receptors exhibited significantly larger, more negative holding currents (-260 ± 40 nA) in comparison to 1:50 (-28 ± 6.0 nA) and 1:100 (-15 ± 5.0 nA) ratios (*p* ≤ 0.001; Tukey’s test; Fig 4A). The application of 100 μM Zn^2+^ generated a reduction in inward current at β3γ2L (1:15) receptors (Fig 4B). In contrast, 100 μM Zn^2+^ did not have any effect at β3γ2L (1:50) and (1:100) receptors.

Despite the difference in maximal efficacy (Fig 4C), 2’MeO6MF showed similar potency at all ratios (EC_50_ = 45 μM (1:15), 62 μM (1:50), 63 μM (1:100); *p* > 0.05 for all logEC_50_ comparisons; Tukey’s test; Fig 4D and Table 2). The potency of 2’MeO6MF measured at α2β3γ2L receptors is also not significantly different from β3γ2L receptors at all three ratios (*p* > 0.05 for all comparisons; Dunnett’s test). We then measured the GABA potency, and found that β3γ2L (1:15) receptors displayed a monophasic GABA concentration-response curve with low potency and a shallow Hill slope (EC_50_ = 1.4 mM; *n*_H_ = 0.54; *n* = 6; Fig 4E and 4F and Table 2). In contrast, the data of 1:50 and 1:100 ratios were significantly better fitted to a biphasic Hill equation than a monophasic model (*p* < 0.0001 for both ratios; extra sum-of-squares *F* test; Fig 4E and 4F). At 1:50 ratio, GABA has a high-sensitivity component with an EC_50_ of 63 μM and a low-sensitivity component with an EC_50_ of 5.5 mM. The mean EC_50_ values for β3γ2L (1:100) receptors were very similar to those of β3γ2L (1:50) receptors (EC_50_1_: 64 μM; EC_50_2_: 4.0 mM). β3γ2L GABA_A_Rs expressed at 1:50 and 1:100 ratios exhibit nearly identical properties (no statistical significant differences in all aspects; *p* > 0.05), but are very different in comparison to the receptors expressed at 1:15 ratio.

**Table 2:** GABA, 2’MeO6MF, etomidate and propofol concentration-response curve parameters at β3γ2L (1:15), (1:50) and (1:100) GABA_A_Rs derived from curve-fitting procedures.

Ligands		β3γ2L (ratio)^*a*^		
		1:15	1:50	1:100
**GABA** ^*b*^	***I***_**max**_ **± SEM** ^*c*^	780 **±** 220 nA	1000 ± 250 nA	1000 ± 97 nA
	**EC**_**50_1**_ **(95% CI)**	1.4 (0.87–2.2) mM	63 (47–84) μM	64 (49–83) μM
	**EC**_**50_2**_ **(95% CI)**	–	5.5 (1.3–23) mM	4.0 (1.4–11) mM
	***n***_**H_1**_ **± SEM**	0.54 ± 0.040	1.0	1.0
	***n***_**H_2**_	–	1.0	1.0
	**Frac (95% CI)**	–	0.74 (0.67–0.80)	0.71 (0.65–0.77)
	***n***	6	8	9
**2’MeO6MF**	***E***_**max**_ **± SEM** ^*d*^	1.2 ± 0.10	0.086 ± 0.018	0.10 ± 0.020
	**EC**_**50**_ **(95% CI)**	45 (31–64) μM	62 (22–172) μM	63 (27–143) μM
	***n***_**H**_ **± SEM**	1.8 ± 0.42	1.4 ± 0.88	1.6 ± 0.76
	***n***	8	7	6
**Etomidate (-60 mV)**	***E***_**max**_ **± SEM** ^*e*^	20 ± 1.8	5.5 ± 0.80	3.2 ± 0.40
	**EC**_**50**_ **(95% CI)**	8.7 (6.7–11) μM	55 (27–110) μM	43 (25–76) μM
	***n***_**H**_ **± SEM**	2.0 ± 0.32	1.3 ± 0.37	1.5 ± 0.46
	***n***	5	6	7
**Etomidate (-30 mV)**	***E***_**max**_ **± SEM** ^*f*^	9.9 ± 0.84	N.D.^*g*^	2.8 ± 0.50
	**EC**_**50**_ **(95% CI)**	19 (11–31) μM		60 (19–190) μM
	***n***_**H**_ **± SEM**	1.3 ± 0.33		1.2 ± 0.50
	***n***	4		5
**Propofol**	***E***_**max**_ **± SEM** ^*h*^	5.7 ± 0.55	N.D.^*g*^	0.64 ± 0.23
	**EC**_**50**_ **(95% CI)**	85 (33–220) μM		74 (33–170) μM
	***n***_**H**_ **± SEM**	1.4 ± 0.50		2.0 ± 1.2
	***n***	4		4

^*a*^ The total amount of mRNA injected per oocyte was adjusted to be approximately 5 ng for each ratio.

^*b*^ Data of 1:15 ratio were better fitted to a monophasic model. Data of 1:50 and 1:100 ratios were significantly better fitter to a biphasic model (*p* < 0.0001 for both ratios; extra sum-of-squares *F* test). Concentration-response curves are shown in Fig 4F.

^*c*^ Current amplitude elicited by 30 mM GABA.

^*d*^ 300 μM 2’MeO6MF-elicited current normalised against 3 mM GABA response.

^*e*^ 30 μM (for 1:15) and 300 μM (for 1:50 and 1:100) etomidate-elicited current normalised against 3 mM GABA response.

^*f*^ 300 μM etomidate-elicited current normalised against 3 mM GABA response.

^*g*^ N.D. = not determined.

^*h*^ 300 μM propofol-elicited current normalised against 3 mM GABA response.

### Differential activation of β3γ2L receptors with different subunit stoichiometries by general anaesthetics

**Fig 5:**
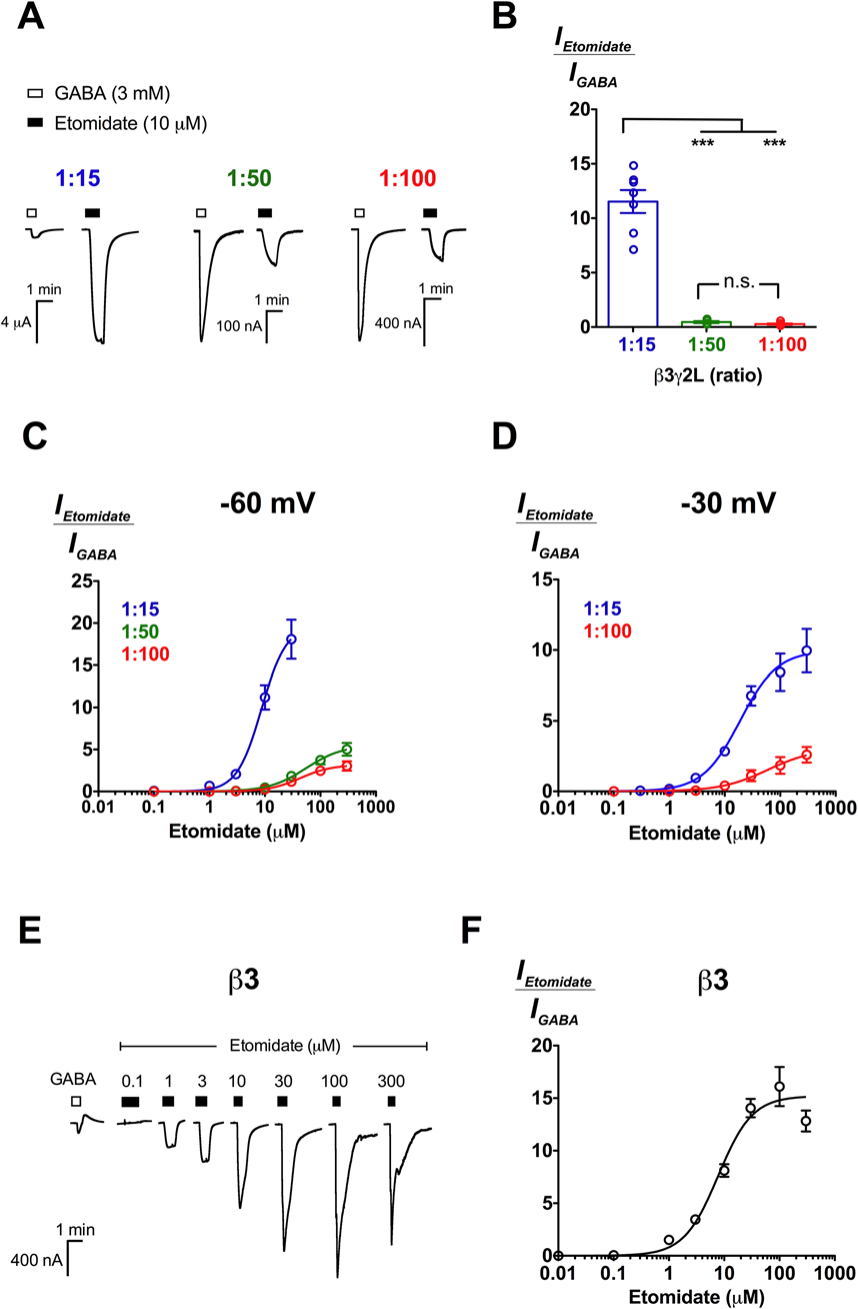
Etomidate activates both β3γ2L and β3 GABA_A_Rs. (A) Representative traces of etomidate (10 μM) direct activation in comparison to 3 mM GABA at β3γ2L (1:15), (1:50) and (1:100) GABA_A_Rs. (B) Mean efficacy of 10 μM etomidate activation at β3γ2L (1:15; *n* = 7), (1:50; *n* = 6) and (1:100; *n* = 7) receptors. Data are normalised to the 3 mM GABA response. The mean efficacy of 10 μM etomidate at various ratios was compared using Tukey’s test, and the significance levels are indicated with n.s. (not significant) and *** (*p* ≤ 0.001). (C) Concentration-response curves of etomidate activation at β3γ2L receptors expressed at 1:15 (*n* = 5), 1:50 (*n* = 6) and 1:100 (*n* = 7) ratios. Recording was conducted at -60 mV. Data are normalised to the 3 mM GABA response. (D) Concentration-response curves of etomidate activation at β3γ2L receptors expressed at 1:15 (*n* = 4) and 1:100 (*n* = 5) ratios. Recording was conducted at -30 mV. Data are normalised to the 3 mM GABA response. (E) Representative traces of β3 homomeric receptors responses to 0.1–300 μM etomidate in comparison to 60 mM GABA response. (F) Etomidate concentration-response curve for β3 homomeric receptors (*n* = 5). Data are normalised against 60 mM GABA responses. Data are presented as mean ± SEM. Bars indicate durations of drug application. The holding current values are represented by the dotted lines.

2’MeO6MF activity has previously been shown to be affected by a well-established mutation that perturbs etomidate activity [30], suggesting that both ligands could share a common activation mechanism. Like 2’MeO6MF, etomidate did not activate homomeric γ2L (Fig 2B) or α2γ2L receptors (*n* = 4; data not shown). However, etomidate activated β3 homomeric receptors efficaciously (*E*_max_ = 15 ± 0.79, normalised against 60 mM GABA), with an EC_50_ of 7.7 μM (95% CI: 5.6–11 μM; *n* = 5; Fig 5E and 5F), in contrast to 2’MeO6MF inhibition of the receptors’ constitutive activity (Fig 2C).

We then evaluated etomidate at β3γ2L (1:15), (1:50) and (1:100) receptors. Etomidate directly activated β3γ2L receptors expressed at all three ratios, albeit with different relative efficacies (Fig 5A). At 10 μM, etomidate directly activated β3γ2L (1:15) approximately 12 ± 1.1 fold higher relative to 3 mM GABA (*n* = 7), significantly greater than at 1:50 (0.46 ± 0.10; *n* = 6) and 1:100 (0.27 ± 0.05; *n* = 7) ratios (*p *≤ 0.001; Tukey’s test; Fig 5B). At β3γ2L (1:50) and (1:100) receptors, consistent with the almost identical GABA and 2’MeO6MF profiles, etomidate activated β3γ2L receptors at both ratios with no significant differences in potencies and maximal efficacies (*p* > 0.05; unpaired *t* test; Fig 5C and Table 2). At 1:15 ratio, we could not construct a complete concentration-response curve as the current elicited by etomidate at concentrations above 10 μM frequently exceeded the measuring range of the amplifier (> 10 μA).

To investigate etomidate activity across the whole concentration range at β3γ2L (1:15) receptors, we conducted electrophysiological recording at -30 mV. The same experiment was repeated at β3γ2L (1:100) receptors as a control. As expected from the voltage-dependent profile of etomidate [41], we observed reduced etomidate activation at -30 mV. However, etomidate still exhibited significantly larger relative efficacy at 1:15 (9.9 ± 0.84) than 1:100 (2.8 ± 0.50) ratio (*p* ≤ 0.01; unpaired *t* test; Fig 5D; Table 2). Etomidate also showed a modest, but significantly higher potency at 1:15 (EC_50_ = 19 μM) than 1:100 (EC_50_ = 60 μM) ratio (*p* ≤ 0.01 for logEC_50_ comparison; unpaired *t* test; Table 2).

**Fig 6:**
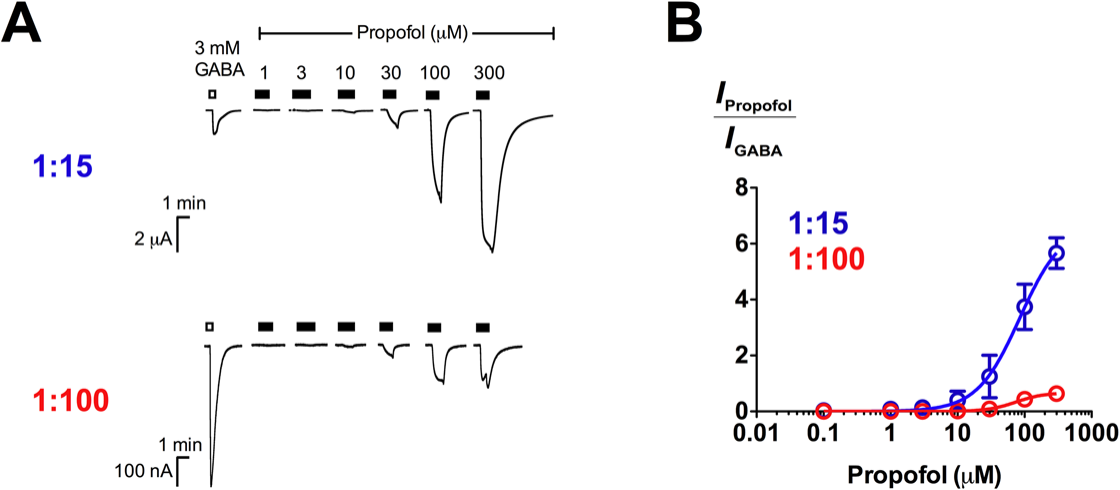
Propofol activates β3γ2L (1:15) and (1:100) receptors with different relative efficacies. (A) Representative traces of propofol (1–300 μM) direct activation in comparison to 3 mM GABA at β3γ2L (1:15) and (1:100) GABA_A_Rs. (B) Concentration-response curves of propofol activation at β3γ2L receptors expressed at 1:15 (*n* = 4) and 1:100 (*n* = 4) ratios. Data are normalised to the 3 mM GABA response.

We also investigated the action of propofol, a non-selective general anaesthetic which has been shown to bind with similar affinity to at least 4 binding sites at GABA_A_Rs found on β+α−, α+β−, β+β− and γ+β− subunit interfaces [42, 43]. We found that propofol directly activated β3γ2L receptors, in agreement with previous findings reported on β2γ2S receptors [26]. Like etomidate, propofol directly activated β3γ2L (1:15) and (1:100) receptors differentially (Fig 6A). At 300 μM, propofol elicited current responses approximately 6-fold larger than maximal GABA currents at β3γ2L (1:15) receptors, significantly larger than at β3γ2L (1:100) receptors (*E*_max_ = 0.64 ± 0.23; *p* ≤ 0.001; unpaired *t* test; Fig 6B; Table 2). However, the potencies of propofol measured at 1:15 (EC_50_ = 85 μM) and 1:100 (EC_50_ = 74 μM) ratios were not different (*p* > 0.05; unpaired *t* test; Table 2).

## Discussion

2’MeO6MF has been previously demonstrated to directly activate recombinant α2β2/3γ2L GABA_A_Rs expressed in *Xenopus* oocytes [30], but the functional significance of each subunit remains unclear. In this study, we performed a systematic investigation of 2’MeO6MF activity at all receptor combinations expressed in *Xenopus* oocytes using α2, β3 and γ2L subunits and found that 2’MeO6MF exhibits distinct pharmacology at β3, β3γ2L and α2β3γ2L GABA_A_Rs. It acts as an inverse agonist at β3 homomers by inhibiting the constitutive activity of the receptors, and activates β3γ2L and α2β3γ2L GABA_A_Rs with different relative efficacies. The lack of activity of 2’MeO6MF at α2β3 (20:1) binary receptors, despite the presence of the β3 subunit, favours an interfacial 2’MeO6MF binding site(s) containing the β3 subunit (β3-β3, β3-γ2L or γ2L-β3) over an intra-β3-subunit binding site.

The robust formation of the non-α-containing β3γ2L GABA_A_Rs raises a critical question of where GABA binds to activate this receptor as it lacks the β+α− interfaces. Despite the absence of any known GABA binding sites in β3γ2L receptors, GABA elicited robust responses within a similar concentration range that activates αβ and αβγ receptors. Thus, we infer that GABA binding site(s) which are previously unknown may exist. A GABA binding site at the β-β interface is possible, but the poor GABA activity at β3 homomeric receptors even at saturating concentrations suggests that other subunit interfaces, possibly between the β and γ subunits are more likely to contribute to the GABA sensitivity of β3γ2L receptors.

There is a wealth of evidence supporting two β+α− interfacial binding sites for etomidate in the transmembrane domain [11–13]. Our data show that etomidate activates β3 homomeric and β3γ2L binary receptors efficaciously, but is insensitive at γ2L homomeric receptors. These findings indicate that the β, but not the α subunit is essential for etomidate sensitivity. This is supported by findings from a photoaffinity labelling study which suggested that etomidate can also bind to a homologous β+β− interfacial site with less selectivity [43]. Besides that, mutations found on the β subunit such as N265M [33] and M286W [44] have been shown to completely eliminate etomidate sensitivity, while mutations found on the α subunit tend to have less profound effects [45]. Taken together, we infer from these findings that the β subunit, which bears the principal (+) components of the etomidate binding sites, dominates ligand binding interactions, whereas the identity of the complementary (−) subunit (α, β or γ) is well tolerated.

We also demonstrate that β3γ2L receptors expressed at different injection ratios exhibit distinct levels of constitutive activity, relative efficacies of 2’MeO6MF, etomidate and propofol activation as well as different GABA potencies. These findings strongly suggest that β3γ2L receptors with different subunit stoichiometries are being formed. The influence of subunit cDNA or mRNA ratios on receptor stoichiometry expressed in heterologous systems is well established in the case of α4β2 nicotinic acetylcholine receptors, in which α4-biased and β2-biased ratios are routinely used to selectively express the pharmacologically dissimilar 3(α4):2(β2) and 2(α4):3(β2) stoichiometries respectively [46, 47]. It is not clear from our experiments which stoichiometries of β3γ2L receptors exist. However, as the binary αβ GABA_A_Rs have been shown to assemble into two functional stoichiometries of 2α:3β and 3α:2β [6, 21, 22], by analogy, we propose that β3γ2L GABA_A_Rs also exist in two different stoichiometries of 3β:2γ and 2β:3γ. It is likely that when the γ2L subunit mRNA was injected in greater abundance relative to the β3 subunit (e.g., 1:50 and 1:100 ratios), the 2β:3γ stoichiometry is predominantly expressed. In contrast, when the injection ratio was reduced (e.g., 1:15), the 3β:2γ stoichiometry may be favourably expressed. The definitive mechanisms underlying the distinct pharmacological profiles of β3γ2L stoichiometric forms are beyond the scope of this investigation, but differences in the number of functional binding interfaces and intrinsic activation properties may have a role. Further experiments are required to understand how these factors result in the complex pharmacology observed for β3γ2L receptors.

We and others have shown that 2’MeO6MF, etomidate, pentobarbital and dopamine appear to be more efficacious agonists than GABA at β3γ2 receptors [27, 29]. In contrast, these ligands directly activate αβγ receptors with lower efficacies relative to the maximal GABA responses [29, 44, 48]. Similar observations have been reported at αβδ receptors, where GABA acts as a partial agonist with low intrinsic efficacy, and modulators such as neurosteroids [49], etomidate [50], propofol [51] and pentobarbital [48] are able to enhance maximal GABA currents significantly larger than they do at αβγ receptors, where GABA acts as a full agonist. It is possible that GABA also acts as a weak partial agonist at βγ receptors. However, this inference is not conclusive as the intrinsic efficacies of GABA and other ligands at βγ receptors are not well established and were not systematically explored in our study. Further studies are required to address these issues, and to clarify whether the apparent larger relative efficacies of various ligands at βγ receptors are due to subtype-dependent functional differences or the low GABA efficacy at these channels.

The expression of βγ receptors has been reported previously [23–29]. In these studies, the overall pharmacological profiles of βγ receptors are very similar to that of the αβγ receptors. This is concerning as pharmacological tools such as diazepam, which is routinely used as an indicator of the purity of the αβγ receptors heterologously expressed would not be able to distinguish βγ from αβγ receptors due to its comparable actions at both receptor subtypes [23, 25]. The GABA_A_R competitive antagonist bicuculline has recently been suggested to have β3γ2-selective pharmacology as it exhibits inhibitory action at α1β3γ2 but activates β3γ2 receptors [29]. However, as bicuculline has been shown to also activate β3 homomers [39], it is not known if the effect observed at β3γ2 receptors was mediated by a background population of β3 homomers. The great diversity in GABA_A_R subtypes dictates the need for subtype-specific ligands to understand the function of these receptors. As such, the search for βγ-selective ligands is pivotal to accurately determine the pharmacology of these receptors *in vitro*.

There is emerging evidence to suggest that native GABA_A_R subtypes are far more diverse than earlier studies have indicated, as exemplified by the *in vivo* expression of αβ receptors which were previously thought to only form in heterologous systems [52–56]. In single channel recording experiments conducted in dissociated rat hippocampal neurons, it has been shown that the activity of 2’MeO6MF, like diazepam, is significantly attenuated by the γ2(381–403) MA peptide which disrupts protein-receptor interactions involving only the γ2 subunit [30, 57]. This finding suggests that γ2-containing GABA_A_Rs are the molecular target of 2’MeO6MF, and the subunit composition of these receptors is most likely to make up of α, β and γ subunits since they are the most common native isoforms. It is currently not known if βγ receptors exist in neurons and thus contribute partially to the flavonoid activation observed. Again, βγ-selective ligands will be necessary to explore this possibility.

## Conclusions

Overall, our findings have important implications in the study of GABA_A_Rs. We have shown that β3γ2L receptors assemble with different subunit stoichiometries in *Xenopus* oocytes, which can be differentiated by GABA, 2’MeO6MF, etomidate and propofol. These receptors lack the α subunit which contributes the complementary (−) components of the GABA and etomidate binding sites, but are still activated by GABA and etomidate, suggesting that the structural requirements for these sites need to be redefined. While the *in vivo* existence of βγ receptors remains elusive, it can serve as a tool to study the contribution of different subunits or subunit interfaces in the pharmacology of a chemical of interest.
